# An early screening model for preeclampsia: utilizing zero-cost maternal predictors exclusively

**DOI:** 10.1038/s41440-023-01573-8

**Published:** 2024-02-07

**Authors:** Lei Wang, Yinyao Ma, Wenshuai Bi, Chenwei Meng, Xuxia Liang, Hua Wu, Chun Zhang, Xiaogang Wang, Hanlin Lv, Yuxiang Li

**Affiliations:** 1 BGI Research, Shenzhen, 518083 China; 2 Guangdong Bigdata Engineering Technology Research Center for Life Sciences, BGI Research, Shenzhen, 518083 China; 3 Department of Obstetrics, People’s Hospital of Guangxi Zhuang Autonomous Region, Nanning, 530021 China

**Keywords:** Preeclampsia, Early screening, Machine learning, Zero-cost predictors, Data augmentation

## Abstract

To provide a reliable, low-cost screening model for preeclampsia, this study developed an early screening model in a retrospective cohort (25,709 pregnancies) and validated in a validation cohort (1760 pregnancies). A data augmentation method (α-inverse weighted-GMM + RUS) was applied to a retrospective cohort before 10 machine learning models were simultaneously trained on augmented data, and the optimal model was chosen via sensitivity (at a false positive rate of 10%). The AdaBoost model, utilizing 16 predictors, was chosen as the final model, achieving a performance beyond acceptable with Area Under the Receiver Operating Characteristic Curve of 0.8008 and sensitivity of 0.5190. All predictors were derived from clinical characteristics, some of which were previously unreported (such as nausea and vomiting in pregnancy and menstrual cycle irregularity). Compared to previous studies, our model demonstrated superior performance, exhibiting at least a 50% improvement in sensitivity over checklist-based approaches, and a minimum of 28% increase over multivariable models that solely utilized maternal predictors. We validated an effective approach for preeclampsia early screening incorporating zero-cost predictors, which demonstrates superior performance in comparison to similar studies. We believe the application of the approach in combination with high performance approaches could substantially increase screening participation rate among pregnancies.

**Figure:**
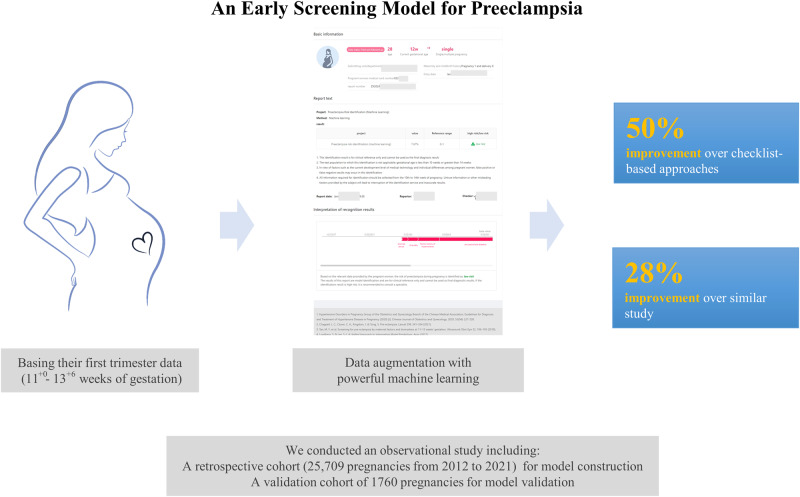
Machine learning model for early preeclampsia screening, using 16 zero-cost predictors derived from clinical characteristics, was built on a 10-year Chinese cohort. The model outperforms similar research by at least 28%; validated on an independent cohort.

Machine learning model for early preeclampsia screening, using 16 zero-cost predictors derived from clinical characteristics, was built on a 10-year Chinese cohort. The model outperforms similar research by at least 28%; validated on an independent cohort.

## Introduction

With a worldwide [1] decline in fertility rates coupled with an increase in childbearing age, early screening for preeclampsia (PE) is garnering increasing attention. Aside from terminating the pregnancy, the existing interventions are only available either before or in early stages of pregnancy [2]. Therefore, PE risk screening is of particular importance for pregnancies as early as possible.

In clinical practice, checklist-based approaches (recommended by American College of Obstetricians and Gynecologists [3] (ACOG) 2018 and National Institute for Health and Clinical Excellence [4] (NICE) 2019) have already been widely adopted as cost-effective but exhibit limited performance [5]. Other multi-variable approaches (Fetal Medicine Foundation (FMF) and others) combining multifactor (including maternal factors, uterine artery pulsatility index (UTPI), mean arterial pressure (MAP), serum placental growth factor (PLGF), and some other biomarkers like cell-free RNA [6, 7] (cfRNA)), have significantly improved prediction performance compared to the checklist-based approach [8]. However, these are limited by the extra costs for (1) facility: standardized training for professionals (such as new measurement and unbiased understanding of involved predictors) and (2) individual: typically uncovered by routine prenatal care, and the latter one constrains the willingness of pregnancies to participate. Therefore, it’s fundamentally important to develop a low-cost approach (both for facilities and individuals) for primary screening that could engage mass pregnancies.

Previous studies reveal that modeling via real-world early pregnancy data would encounter severe challenges: (1) the association between features and outcomes is unclear and non-linear, which needs powerful machine learning (ML) algorithm; (2) low PE incidence rate (typically <8%) (namely class-imbalance) could lead to overfitting in ML modeling or lack of generalizability for further clinical application, which needs suitable data augmentation (DA) methods [9].

Traditional DA methods [10] like Random Under Sampling (RUS) and Random Over Sampling (ROS) resolve class-imbalance but neglect the original data diversity. Methods like the Synthetic Minority Over-sampling Technique (SMOTE) and its variants could perform well if it applied to datasets with clear class boundaries, but for early pregnancy data, their effectiveness is limited due to their exclusive reliance on spatial distance [11]. In this study, we intend to utilize a DA method [12] with proven effectiveness, that combines both the Gaussian Mixture Model (GMM) for generating new positive samples and under-sampling to adjust class ratios.

By leveraging suitable DA methods, powerful ML algorithms and 10-years duration training data, we aimed to conduct an early screening model for PE. We emphasize the use of ‘zero-cost’ predictors, which are variables readily available from routine prenatal care that do not incur additional expenses. This approach enhances the accessibility and feasibility of our predictive model, making it an ideal tool for widespread application in natural population settings.

## Methods

### Study population

This was a single-center, observational, real-world study, involving a retrospective study for model construction and a validation study to assess its clinical applicability. All studies were held at the People’s Hospital of Guangxi Zhuang Autonomous Region in China.

The retrospective cohort included pregnancies that attended prenatal care at 11^+0^ - 13^+6^ weeks of gestation between April 2012 and Sep 2021, and excluded that either (1) ended in termination, miscarriage, or fetal death before 24 weeks of gestation; or (2) had no delivery record.

After model construction we observed and included pregnancies that attended prenatal care at 11^+0^ - 13^+6^ weeks of gestation from Sep 2021 to Sep 2022 into the validation cohort, and the exclusions were the same as retrospective cohort, which (1) ended in termination, miscarriage, or fetal death before 24 weeks of gestation or (2) without delivery record.

It’s important to clarify that the validation study was also conducted retrospectively.

### Outcome measures

We initially assigned all pregnancies with “PE” or “non-PE” label according to diagnosis extracted from delivery reports, and the labels were rule-reviewed by clinical experts to ensure reliability. The review rule was established according to the diagnostic criteria of PE which is high blood pressure (systolic blood pressure ≥ 140 mmHg or diastolic blood pressure ≥ 90 mmHg) accompanied by proteinuria after 20 weeks of gestation [13].

For further analysis, we identified cases of ‘preterm-PE’ for deliveries with PE before 37 gestational weeks and ‘early onset-PE’ for those before 34 gestational weeks.

### Data processing and feature selection

Our study tended to utilize ‘zero-cost’ predictors that were routinely accessible from standard prenatal care and with established benchmarks [14] in previous research. Hence, we paid more attention on domains of maternal demographic information, obstetric history, menstrual details, medical history, drug allergy history, delivery report, family medical history and lab tests for feature extraction. Besides, we included the medical history of the biological father of the fetus.

Lab tests conducted before the 14th gestational week were considered, excluding those with over 80% missing values on whole cohort. Each test was categorized into ‘normal’ or ‘abnormal’ based on the corresponding reference range, and missing values were imputed with ‘uncertain’.

Before feature selection, we applied one-hot encoding to all categorical features and performed statistical analysis using Mann Whitney *U* test and ${\chi }^{2}$ test for continuous and categorical features, respectively. Candidate features were those with a *p* value < 0.05. Employing random forest (RF) as basic classifier, we assessed feature importance through 5-fold cross-validation. Features were ranked based on their mean importance, with a cut-off established at the inflection point of a cumulative importance exceeding 0.80. Features above this cut-off were included as predictors in the training dataset for further model construction.

### Data augmentation

We employed three kinds of DA methods, GMM + RUS and its variants (α-inverse weighted-GMM + RUS, inverse weighted-GMM + RUS) to tackle class-imbalance in training dataset. With the help of Individual Bayes Imbalance Impact Index (IBI^3^) and the Bayes Imbalance Impact Index (BI^3^) [15], we could independently assess the improvement of the above methods and choose the optimal one. Typically, lower BI^3^ values and IBI^3^ variances indicate that the classifier is less affected by class-imbalance. Detailed process is listed in the appendix.

### Model construction and validation

To develop our predictive model, we constructed several ML models known for effectiveness in similar tasks. These included Adaptive Boosting (AdaBoost) [16] and various other algorithms. We optimized each model using standard techniques like grid search and cross-validation, with Area Under the Curve (AUC) as the primary performance metric.

Model performance was assessed in the internal validation set, focusing on sensitivity at a false positive rate (FPR) of 10% (equivalent to a specificity of 90%) and AUC. AdaBoost, demonstrating the highest sensitivity, was selected for further external validation. More details about the model selection process, hyperparameter optimization, and comprehensive evaluation are available in the appendix.

The external validation involved assessing the risks of PE using the AdaBoost model and comparing its performance against established guidelines like NICE 2019 and ACOG 2018, as detailed in the appendix.

### Software packages

The code, developed to support the findings of this study, was specifically designed for and tailored to the structure of the hospital’s database and its inherent data. While the full utility of the code is limited without access to the corresponding data, a portion of the code, particularly for model construction, has been made publicly accessible to facilitate research transparency and reproducibility. This shared code is available on GitHub[https://github.com/dctongsheng/An-Early-Screening-Model-for-Preeclampsia-Utilizing-Zero--Cost-Maternal-Predictors-Exclusively.git]. All data processing and modeling tasks were performed using Python 3.8, employing publicly accessible standard libraries: pandas, numpy, sklearn, imblearn, matplotlib, xgboost, lightgbm, catboost and shap.

## Results

### Study population characteristics

The retrospective study initially included 31,384 pregnancies, and after the exclusion (most without delivery reports), 25,709 pregnancies were incorporated into the retrospective cohort, among which 1635 (6.36%) were PE, including 612 (2.49%) preterm-PE cases and 285 (1.11%) early onset-PE cases.

For the validation cohort, we initially involved 1796 pregnancies. After removing 33 pregnancies without delivery reports and 3 pregnancies that resulted in miscarriage before 24 weeks of gestation, a total of 1760 pregnancies were included in the final analysis. Among these, 158 (8.97%) were PE, with 62 being preterm-PE (3.52%) and 27 (1.53%) early onset-PE cases.

Our study was conducted at a provincial-level obstetric referral center, and we observed a decade-long increasing PE incidence, with a notable surge (13.4%) in 2020. While none of the pregnant women in our dataset were diagnosed with COVID-19 in 2020, suggesting no direct link to the pandemic, discussions with experts suggest that the relative concentration of patients could be associated with pandemic control policies.

### Feature selection

Our study encompassed more potential predictors than previous studies. A total of 43 clinical characteristics and 148 lab features were extracted from electronic medical records. All clinical characteristics are reported in Supplementary Tables 1 and 7.

**Fig. 1:**
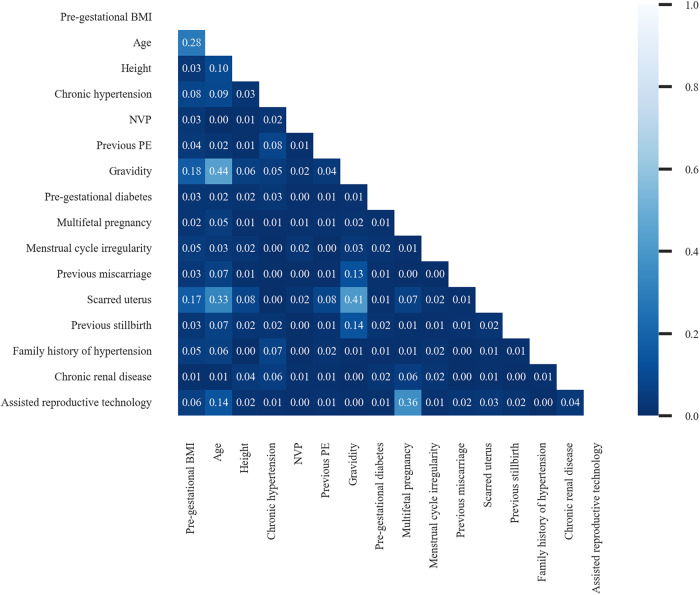
Correlation of 16 predictors. This figure presents pairwise correlation between all predictors. Almost all predictors demonstrated linear independence from each other (with correlations less than 0.1). Some special pairs (such as age and gravidity, gravidity and scarred uterus, assisted reproductive technology and multifetal pregnancy) do exhibit certain linear correlations. However, these are easily understandable based on common sense

After statistical analysis, all candidate features (*p* value < 0.05) were then ranked in descending order by their mean importance to RF-classifier. An examination of the cumulative importance revealed a suitable inflection point at approximately 0.85, which was selected as cut-off point. After all, a total of 16 predictors were chosen, which are listed in Supplementary Table 2. The correlation of predictors is illustrated in Fig. 1.

### Data augmentation

**Fig. 2:**
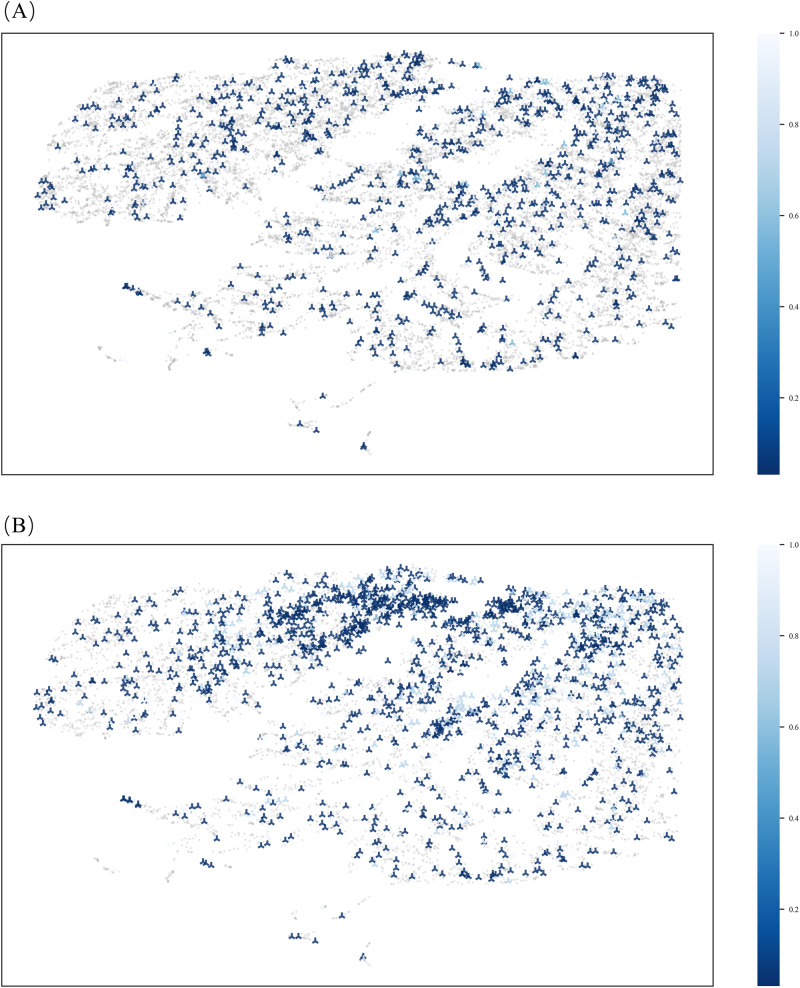
UMAP diagrams representing sample distribution with corresponding IBI^3^ before and after DA. This figure presents the UMAP diagrams of all samples in the training dataset (**A**) before and (**B**) after DA. PE cases are denoted by crosses, with color intensity ranging from blue (0) to no color (1), indicating the corresponding IBI^3^ scores. The IBI^3^ value reflects the degree of imbalance impact on a minority (positive) class sample, with a value closer to 1 indicating a higher class-imbalance impact for model and a potential improvement could be achieved

As shown in Fig. 2A, a UMAP overview of the training dataset reveals that the distribution of inter or intra class samples are highly overlapping, and the average Euclidean distances between classes, as well as the intra-class average distance among positive samples, are nearly identical, at 10.16 and 10.43 respectively (*p* value = 0.73, indicating no significant difference between the inter and inter distance).

Upon comparing three methods (GMM, inverse weighted-GMM, α-inverse weighted-GMM), we found the α-inverse weighted-GMM + RUS to be the most effective in dealing with class imbalance. This method yielded the highest improvement in BI^3^ (38.7%, compared with 30.87% of inverse weighted-GMM + RUS and 6.1% of GMM + RUS). For its parameter, we chose the optimal GMM components to 15, fine-tuned the α value to 1.84, and adjusted the positive-to-negative ratio to 1:3. Detailed improvement distribution is illustrated in Fig. 2.

### Model performance

**Fig. 3:**
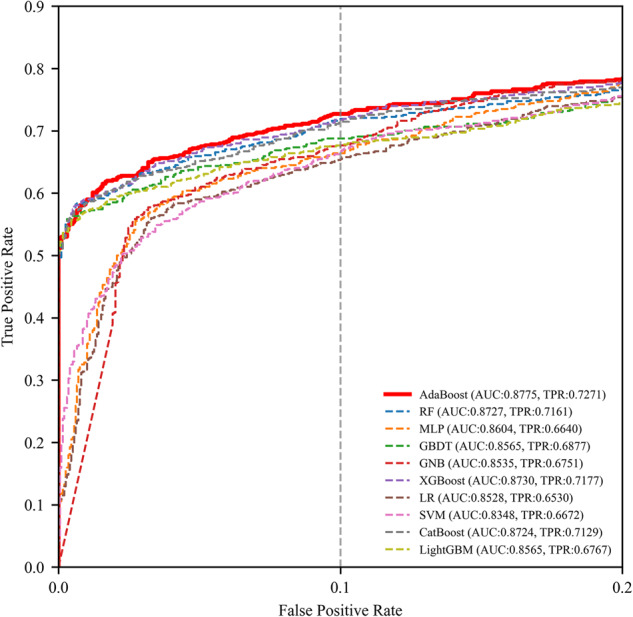
Comparison of ROC curves for 10 ML models. This figure presents collective ROC curves of the 10 ML models in the internal validation set, providing an overview of their performances with a focus on FPR in [0, 0.2]. It’s obvious that the AdaBoost outperforms all models

The performance metrics of 10 models in internal validation set are summarized in Supplementary Table 3, with ROC curves depicted in Fig. 3 and Supplementary Fig. 2. Notably, the AdaBoost model demonstrated best performance, achieving a sensitivity of 0.7271 (95% CI, 0.6924–0.7619) at a 10% FPR and an AUC of 0.8775 (95% CI: 0.8612–0.8942). Thus, the 16-predictor-AdaBoost-model was selected as the final model.

**Fig. 4:**
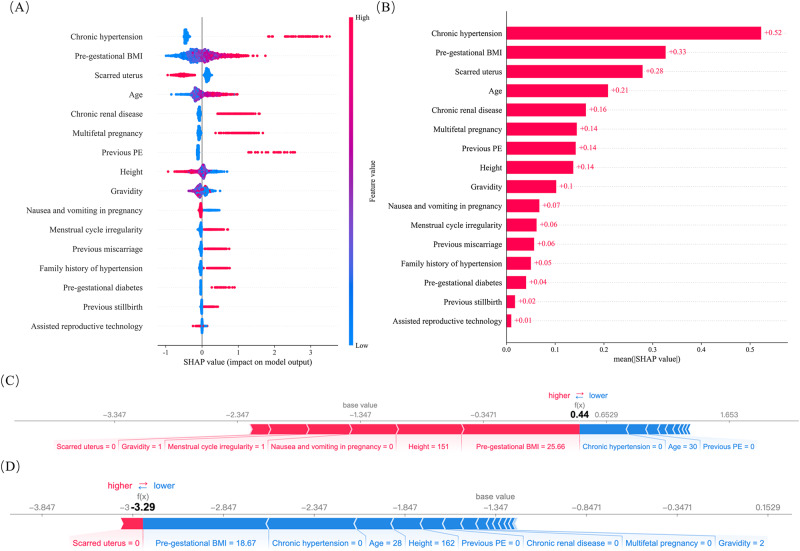
Feature importance and contribution analysis. **A** SHAP summary plot for the finial model, presenting SHAP values for individual pregnancies across 16 predictors, arranged in descending order of mean absolute SHAP value. The color of each point denotes the predictor’s value. **B** A ranking of predictors based on their average absolute impact on the AdaBoost model output. Composition of predictors for an example PE patient (**C**) and a normal pregnancy (**D**). Red and blue arrows denote the influences of individual predictors that increase and decrease the likelihood of developing PE, respectively

In external validation, the AdaBoost model demonstrated an AUC of 0.8008, a sensitivity of 0.5190 at a FPR of 10%. For preterm-PE, this model achieved an AUC of 0.8164 and a sensitivity of 0.5323 at a 10% FPR. In the case of early onset PE, the AdaBoost model showed a sensitivity of 0.5815 and an AUC of 0.815 at a 7% FPR. Detailed performance is listed in Supplementary Tables 4 and 5. A detailed SHAP analysis, illustrated in Fig. 4, identified chronic hypertension as a significant predictor.

In benchmark comparison, our model displayed a sensitivity of 0.3734 compared to the NICE 2019 of 0.2346 at equivalent FPRs, and 0.4051 versus the ACOG 2018 of 0.2928, as detailed in Table 1.

**Table 1:** Model performance comparison with previous studies

Study	Internally validated		Externally validated		Feature groups				
	AUC	Sensitivity	AUC	Sensitivity	Maternal factors	MAP	Biochemical markers	Doppler	Lab tests
***Checklist-based approaches comparison***									
NICE 2019 [4] (all-PE)^a^	–	–	–	0.23	**✓**				
Our model (all-PE)^a^	–	–	0.80	0.37	**✓**				
ACOG 2018 [3] (all-PE)^b^	–	–	–	0.29	**✓**				
Our model (all-PE)^b^	–	–	0.80	0.41	**✓**				
***Multivariable approaches comparison***									
Our model (all-PE)^c^	0.88 (0.86, 0.89)	0.73 (0.69, 0.76)	0.80	0.52	**✓**				
Our model (preterm-PE)^c^	–	–	0.82	0.53	**✓**				
Our model (early onset-PE)^d^	–	–	0.82	0.58	**✓**				
Wright et al. [25] (all-PE)^c^	0.76	0.40 (0.39–0.42)	–	–	**✓**				
Wright et al. [25] (preterm-PE)^c^	0.79	0.48 (0.44–0.51)	–	–	**✓**				
Wright et al. [25] (early onset-PE)^c^	0.81	0.54 (0.48, 0.59)	–	–	**✓**				
Wright et al. [26] (all-PE)^c^	0.83 (0.81,0.84)	0.52 (0.49,0.55)	0.85 (0.83,0.87)	0.53 (0.49,0.58)	**✓**	**✓**	**✓**	**✓**	
Wright et al. [26] (preterm-PE)^c^	0.91 (0.89,0.93)	0.75 (0.70,0.80)	0.93 (0.92,0.95)	0.83 (0.76,0.89)	**✓**	**✓**	**✓**	**✓**	
Wright et al. [26] (early onset-PE)^c^	0.95 (0.93, 0.97)	0.87 (0.80, 0.92)	0.96 (0.93, 0.98)	0.90 (0.78, 0.96)	**✓**	**✓**	**✓**	**✓**	
O’Gorman et al. [39] (preterm-PE)^c^	0.80	0.49 (0.43–0.55)	–	–	**✓**				
O’Gorman et al. [39] (preterm-PE)^c^	0.91	0.75 (0.70–0.80)	–	–	**✓**	**✓**	**✓**	**✓**	
Marić et al. [20] (all-PE)^e^	0.79 (0.75–0.83)	0.45	–	–	**✓**				**✓**

*PE* Preeclampsia, *AUC* Area Under the Receiver Operating Characteristic Curve, *MAP* Mean Arterial Pressure, *NICE* National Institute for Health and Clinical Excellence, *ACOG* American College of Obstetricians and Gynecologists

^a^At an 3% FPR

^b^At an 5% FPR

^c^At an 10% FPR

^d^At an 7% FPR

^e^At an 8% FPR. All data above retained to two decimal places

### Additional research on chronic hypertension

Chronic hypertension is identified as a significant predictor in our AdaBoost model for PE. Motivated by a desire to evaluate the robustness of our model across different patient groups, particularly in relation to this key predictive factor, we carried out a stratified analysis. This analysis involved separating the patients into two subgroups: those with chronic hypertension and those without.

In our findings, both subgroups exhibited a modest decrease in model performance. Specifically, the subgroup with chronic hypertension showed a sensitivity of 0.3414 and an AUC of 0.6564, while the subgroup without chronic hypertension demonstrated a sensitivity of 0.4392 and an AUC of 0.7571. These results, which are further detailed in Supplementary Table 6, indicate that, although performance reduction was observed in both subgroups, the model generally maintains an acceptable level of accuracy in predicting PE.

## Discussion

### Principal findings

Our study extracted some features that were not popular in PE-related analysis and led two noteworthy features into our model: nausea and vomiting in pregnancy (NVP) and menstrual cycle irregularity.

NVP revealed a lower prevalence in the PE group (odds ratio (OR) 0.63, *p* value < 0.001), which potentially supports the hypothesis by Flaxman et al. [17] that nausea and vomiting in pregnancy serves as a defense mechanism for both mother and embryo. However, the case of hyperemesis gravidarum [18–20], a severe form of NVP, reported a positive association with PE. Chortatos et al. [21]. in a Norwegian cohort revealed slight differences between NVP and nausea in pregnancy, with association with PE (OR 1.13 and OR 0.83, respectively). These findings emphasize the potential value of additional cohort studies, such as those focused on specific NVP types and PE.

Another higher incidence of menstrual cycle irregularity was observed in the PE group (OR 1.85, *p* value < 0.001). Although we did not find any direct reports linking menstrual cycle irregularity with PE, existing studies have reported its associations with some recognized risk factors, such as chronic hypertension and obesity. Specifically, Chung et al. [22]. and Rostami et al. [23]. found a strong association between menstrual cycle irregularity and chronic hypertension, and Harlow et al. [24]. documented that a menstrual cycle longer than 43 days was associated with being 15% overweight, which resulted in higher pre-pregnancy body mass index (BMI). The direct association between menstrual cycle irregularity and PE still requires further in-depth research.

Despite our initial consideration of a broad array of lab tests and established predictors, such as smoking history, systemic lupus erythematosus (SLE), antiphospholipid syndrome (APS), and racial origin, none were included in our final model for various reasons.

Though the prevalence of SLE and APS and the association with PE in our study were quite similar with previous studies (OR 2.50 with a *p* value of 0.005, compared with OR 2.98 with a *p* value < 0.001 [25]), but it was excluded from final model as its mean importance was lower than 0.02 and failed to contribute the model. In addition, the incidence of smoking history and the association with PE were notably low in our dataset (0.05%, with a *p* value of 0.7) compared with previous studies (9.7% with a *p* value < 0.001 [25], 9.08% with a *p* value < 0.05 [26]). The childbearing practices in China tend to protect the pregnancies by making them less exposed to the effects of smoking, including the suppression of their life partners and family members [27].

In this study, we introduced a new feature “Ethnicity”, which is a subcategory of racial origin, and a significant disparity in PE prevalence was observed with the PE group comprising a higher proportion of minority ethnic groups. As we know, there are 56 ethnic groups in China, and our cohort only encompassed Han, Zhuang, Yao, and Miao. Hence, even ethnicity might optimize our model’s performance (by 5.8% in sensitivity in validation dataset), it was excluded to ensure full generalizability.

### Comparison with previous studies

We evaluated the performance of our model against two prevalent screening approaches: checklist-based methods and multivariable models, as detailed in Table 1. This evaluation included assessments for all-PE, preterm-PE, and early onset PE.

We applied the ACOG 2018 and NICE 2019 guidelines to our external dataset, and a head-to-head performance comparison demonstrated our model’s superior performance, with a minimum increase of 50% in sensitivity at equivalent FPRs.

For checklist-based approach, we applied recommendations from ACOG and NICE with our external dataset, enabling a head-to-head performance comparison. Our model achieved beyond acceptable performance, which by a minimum of 50% improvement in sensitivity over checklist-based approaches (at a FPR of 2.87% with NICE and 4.94% with ACOG).

As multivariable studies seldom released their datasets and models, we were compelled to a direct comparison via reported performance. Our model demonstrated a significant increase of 28% in sensitivity (0.519 versus the highest reported sensitivity of 0.403 [25], at a FPR of 10%), and an improvement of approximately 9.7% in sensitivity with ML model integrating lab tests (0.4936 versus 0.452 [20], at an FPR of 7.9% versus 8.1%) in predicting all-PE. Interestingly, while our model utilizes zero-cost predictors require no additional tests or financial expenditure, its performance was found to be commensurate with some that utilized more advanced predictors, such as MAP, PLGF, and others. These results further substantiate the performance of our study for early screening.

### Limitations

Our study’s retrospective nature over the past decade introduced several challenges in data collection, particularly concerning the completeness and reliability of certain variables: 1. The blood pressure measured before 14 gestational weeks extracted were single measurements taken during prenatal care visits. The lack of context, such as whether these were bilateral averages, precluded the calculation of a reliable MAP, limiting their utility in our analysis; 2. While family history of PE is often cited as a risk factor, it was not included in our model. In contrast to other family medical histories like diabetes or cancer, which tend to be better documented, the specific diagnosis of PE during pregnancy in previous generations was less reliably recorded. This inconsistency in documentation led us to exclude PE family history from our predictive model; 3. The inclusion of lab test results was hindered by a high missing value ratio. Despite the potential value of additional features (such as thyroid-stimulating hormone, reticulocyte percentage, urinary protein, and platelet distribution width, each with a *p* value < 0.001), their high missing rates significantly impacted the model’s performance and were, therefore, not included.

Lastly, our study was conducted in a single center, which may limit the generalizability of our findings. Future research should focus on multi-center studies to enhance the model’s applicability across broader clinical settings.

## Conclusions

In this study, we revealed an effective PE screening model in early pregnancy, outperforming previous similar studies. By combining new features and suitable DA, we employed the 10-year observational data to construct a model with good generalizability and robustness. The 16 predictors in our study, which can be accurately understood and self-assessed by pregnancies, offer a zero-cost approach for all pregnancies as primary risk screening tool, even suitable for use at home.

In the future, we propose establishing a secondary mechanism for PE screening. Following primary screening specifically targets a more precise prediction in high-risk patients, the acceptance of advanced predictors such as cfRNA could be potentially encouraged for secondary screening.

### Supplementary information

Supplementary Table 1

Supplementary Table 2

Supplementary Table 3

Supplementary Table 4

Supplementary Table 5

Supplementary Table 6

Supplementary Table 7

Supplementary Figure 1

Supplementary Figure 2

Supplementary Figure legend

## Appendix

**Data augmentation**

In our study, we employed three kinds of DA methods, GMM + RUS and its variants (α-inverse weighted-GMM + RUS, inverse weighted-GMM + RUS) to tackle class-imbalance in training dataset. By using the Individual Bayes Imbalance Impact Index (IBI^3^) and the Bayes Imbalance Impact Index (BI^3^) [15], we were able to independently evaluate the improvement of these methods and select the optimal one.

The computation of IBI^3^ begins by determining the ratio of negative class num (refer to the majority class num,${N}_{n}$) to positive class num (refer to the minority class num, ${N}_{p}$): ${r=N}_{n}/{N}_{p}$. For each positive class sample, the algorithm calculates the num of majority class neighbors (M) using K-Nearest Neighbors (with a setting of K = k_0_). If M = 0, M is replaced with the num of negative class samples between the current sample and its nearest positive class neighbor, and a variable k is set to M + 1. Otherwise, k is set to a predefined num of nearest neighbors (k_0_). The algorithm then calculates the local probabilities of false negatives (${f}_{n}({{{{{\rm{x}}}}}})={{{{{\rm{M}}}}}}/{{{{{\rm{k}}}}}}$), false positives (${f}_{p}({{{{{\rm{x}}}}}})=({{{{{\rm{k}}}}}}-{{{{{\rm{M}}}}}})/{{{{{\rm{k}}}}}}$, and an adjusted false positive rate for the balanced case (${f}_{p}^{{\prime} }\left({{{{{\rm{x}}}}}}\right)={{{{{\rm{r}}}}}}({{{{{\rm{k}}}}}}-{{{{{\rm{M}}}}}})/{{{{{\rm{k}}}}}}$).

The individual Bayes Imbalance Impact (IBI^3^) for each sample is calculated as:

$${{{{{{\rm{IBI}}}}}}}^{3}({{{{{\rm{x}}}}}})={f}_{p}^{{\prime} }({{{{{\rm{x}}}}}})/({f}_{n}({{{{{\rm{x}}}}}})+{f}_{p}^{{\prime} }({{{{{\rm{x}}}}}}))-{f}_{p}({{{{{\rm{x}}}}}})/({f}_{n}({{{{{\rm{x}}}}}})+{f}_{p}({{{{{\rm{x}}}}}}))$$

Finally, the BI^3^ for the whole dataset is obtained by averaging all IBI^3^ values for the positive class. This metric provides an estimate of the overall impact of class imbalance on the dataset. Typically, lower BI^3^ values and smaller variances in IBI^3^ indicate that the classifier is less affected by class-imbalance.

The combination method (GMM + RUS) was utilized in our study. This method generates new positive samples without removing the original ones and under-samples negative samples with acceptable diversity loss.

There are two common derivative applications of GMM: direct GMM and inverse weighted-GMM. The main difference between them lies in the weighting, or ‘weight’, assigned to the GMM components when generating new samples. The initial weights are determined through model fitting, which is based on the original data distribution. Direct GMM employs initial weights directly, while inverse weighted-GMM adopts the normalized reciprocal of initial weights (${w}_{o}$), thus the variant weight, inverse weight ${w}_{i}=1/{w}_{o}$.

Inverse weight places more emphasis on the generation of sparse-space samples, thereby endowing newly generated data with greater diversity. And to maximize data sparsity, we introduced α-inverse weight where the variant weight ${w}_{i}=1/{w}_{o}^{\alpha }$.

For the number of new samples, we empirically assign minimum weight to the original positive sample count, thereby the increment (${N}_{i}$) from original positive samples (${N}_{o}$) is:

$${N}_{i}={N}_{o}* (1-\min {w}_{i})$$

In training dataset, we compared three methods (GMM, inverse weighted-GMM, α-inverse weighted-GMM), and utilized IBI^3^ and BI^3^ metrics to quantify the improvement in model impact in relation to class imbalance. Through comparing the distributions of IBI^3^ and BI^3^ values, we chose the best method and optimal α via lowest BI^3^ value. The α-inverse weighted-GMM exhibited the best performance, detailed in Supplementary Fig. 1A.

To adjust the class ratio, we used the unbiased RUS to reduce the num of negative samples. With BI^3^ scoring, we assessed model performance under various class ratios following α-inverse weighted-GMM via AUC performance. Eventually a 1:3 class ratio was determined to be the optimal value (an BI^3^ of 0.0879).

Supplementary Fig. 1B illustrates a comparison of model performance before and after DA with a 6.5% improvement in sensitivity (at a FPR of 10%) within the validation dataset.

**Model construction and validation**

The training dataset was split into two subsets at a 7:3 ratio: (1) a training set for hyperparameter tuning and model fitting; and (2) an internal validation set for performance comparison.

We constructed 10 models which had proven performance in similar prediction tasks, namely Adaptive Boosting (AdaBoost) [16], RF [28], Multi-Layer Perceptron (MLP) [29], Gradient Boosting Decision Tree (GBDT) [30], Gaussian Naive Bayes (GNB) [16], Extreme Gradient Boosting (XGBoost) [31], Logistic Regression (LR) [32], Support Vector Machines (SVM) [33], Category Boosting (CatBoost) [34], and Light Gradient Boosted Machine (LightGBM) [35]. For each model, we performed hyperparameters optimization individually using grid search [36] and 5-fold cross-validation, with Area Under the Receiver Operating Characteristic Curve (AUC) as the scoring metric.

Models were compared in internal validation set using sensitivity at a false positive rate (FPR) of 10% (equivalent to a specificity of 90%) and AUC. A range of other metrics were also employed to comprehensively evaluate the performance of our predictive models. These included: Positive Predictive Value (PPV), Negative Predictive Value (NPV), F1 Score, Accuracy, Brier Score, Cohen’s Kappa, Matthew’s Correlation Coefficient (MCC). The mean and 95% confidence interval (CI) of these metrics were calculated using bootstrapping. The model with highest sensitivity was chosen for external validation, with the contributions of each predictor quantified via SHapley Additive exPlanations (SHAP) [37, 38].
