# Supplementary material for: An early screening model for preeclampsia: utilizing zero-cost maternal predictors exclusively
Source: Hypertens Res. 2024 Feb 7;47(4):1051–62. doi: 10.1038/s41440-023-01573-8 (PMC10994845; doi:10.1038/s41440-023-01573-8)
Supplement: Supplementary file 1 — Supplementary Table 1 [file 41440_2023_1573_MOESM1_ESM.docx]

### Supplemental Table 1. Clinical characteristics of the training cohort.

| **Feature** | **non-PE**  **(n=24,074)** | **PE**  **(n=1635)** | ***p*-value** |
| --- | --- | --- | --- |
| Weeks of delivery^a^ | 38.40 (1.67) | 36.36 (3.13) | *** |
| Systolic BP at delivery, mmHg^a^ | 122.78 (13.20) | 155.02 (14.67) | 0.12 |
| Diastolic BP at delivery, mmHg^a^ | 74.0 (8.21) | 92.17 (9.85) | *** |
| Pre-pregnancy weight, kg^a^ | 53.67 (8.03) | 57.03 (9.69) | *** |
| Occupation | | | *** |
| Stable | 1784 (7.41%) | 142 (8.69%) |  |
| Unstable | 629 (2.61%) | 74 (4.53%) |  |
| Unrecorded | 21661 (89.98%) | 1419 (86.79%) |  |
| Menstrual Volume | | | 0.0043 |
| Hypomenorrhea | 226 (0.94%) | 20 (1.22%) |  |
| Normal | 23486 (97.56%) | 1574 (96.27%) |  |
| Menorrhagia | 137 (0.57%) | 12 (0.73%) |  |
| Unrecorded | 225 (0.93%) | 29 (1.77%) |  |
| Marital Status | | | 0.1141 |
| Married | 21478 (89.22%) | 1470 (89.91%) |  |
| Unmarried | 671 (2.79%) | 54 (3.3%) |  |
| Unrecorded | 1925 (8.0%) | 111 (6.79%) |  |
| Ethnicity | | | *** |
| Han | 14208 (59.02%) | 799 (48.87%) |  |
| Ethnic Minority | 9866 (40.98%) | 836 (51.13%) |  |
| Age of menarche, y^a^ | 13.28 (1.21) | 13.2 (1.31) | 0.01 |
| Menstrual cycle, d^a^ | 32.75 (8.05) | 33.19 (9.27) | 0.03 |
| Parity^a^ | 1.5 (0.59) | 1.51 (0.64) | 0.88 |
| Smoking history | 13 (0.05%) | 0 (0.0%) | 0.7103 |
| **Maternal Medical history** | | | |
| SLE/APS | 83 (0.34%) | 14 (0.86%) | 0.005 |
| Hepatitis B | 301 (1.25%) | 20 (1.22%) | 1 |
| Placental abruption | 56 (0.23%) | 12 (0.73%) | *** |
| Primigravid | 7760 (32.23%) | 529 (32.35%) | 0.9412 |
| Thalassemia | 2670 (11.09%) | 196 (11.99%) | 0.2826 |
| Cesarean section | 6352 (26.39%) | 399 (24.4%) | 0.0831 |
| Dysmenorrhea | 5126 (21.29%) | 313 (19.14%) | 0.0426 |
| Miscarriage | 1571 (6.53%) | 144 (8.81%) | *** |
| Induced abortion | 9935 (41.27%) | 670 (40.98%) | 0.8379 |
| Ovulation induction | 77 (0.32%) | 8 (0.49%) | 0.3511 |
| Hyperlipidemia | 735 (3.05%) | 51 (3.12%) | 0.9393 |
| Anemia | 349 (1.45%) | 17 (1.04%) | 0.2127 |
| G6PD deficiency | 809 (3.36%) | 49 (3.0%) | 0.471 |
| Surgery history | 12211 (50.72%) | 939 (57.43%) | *** |
| Cancer | 1116 (4.64%) | 92 (5.63%) | 0.0763 |
| Uterine fibroids | 470 (1.95%) | 44 (2.69%) | 0.0763 |
| Endometrial disease | 254 (1.06%) | 35 (2.14%) | *** |
| Fallopian tube disease | 756 (3.14%) | 99 (6.06%) | *** |
| Cardiovascular disease | 132 (0.55%) | 26 (1.59%) | *** |
| Ovarian disease | 784 (3.26%) | 67 (4.1%) | 0.077 |
| Preterm delivery | 131 (0.54%) | 11 (0.67%) | 0.6124 |
| Adverse pregnancy outcomes | 2386 (9.91%) | 226 (13.82%) | *** |
| **Drug allergy history** | 4225 (17.55%) | 311 (19.02%) | 0.1397 |
| Cephalosporin | 289 (1.2%) | 13 (0.8%) | 0.1759 |
| Penicillin | 901 (3.74%) | 59 (3.61%) | 0.8342 |
| Sulfanilamide | 265 (1.1%) | 19 (1.16%) | 0.9146 |
| **Family history** | | | |
| Diabetes | 493 (2.05%) | 41 (2.51%) | 0.2412 |
| Cardiovascular disease | 52 (0.22%) | 8 (0.49%) | 0.051 |
| Cancer | 754 (3.13%) | 71 (4.34%) | 0.0089 |
| **Medical history of the biological father of the fetus** | | | |
| G6PD deficiency | 46 (0.19%) | 0 (0.0%) | 0.1424 |
| Weak sperm | 45 (0.19%) | 4 (0.24%) | 0.8221 |
| Hepatitis B | 225 (0.93%) | 5 (0.31%) | 0.0132 |

**^a^** Data are presented as mean (standard deviation); ***: *p*-value < 0.001.

*PE* Preeclampsia, *BP* Blood Pressure, *SLE* Systemic lupus erythematosus, *APS* antiphospholipid syndrome, *G6PD* Glucose-6-Phosphate Dehydrogenase.
