# Supplementary material for: An early screening model for preeclampsia: utilizing zero-cost maternal predictors exclusively
Source: Hypertens Res. 2024 Feb 7;47(4):1051–62. doi: 10.1038/s41440-023-01573-8 (PMC10994845; doi:10.1038/s41440-023-01573-8)
Supplement: Supplementary file 2 — Supplementary Table 2 [file 41440_2023_1573_MOESM2_ESM.docx]

### Supplemental Table 2. Statistic of predictors on retrospective and validation cohorts.

| **Predictor** | **Retrospective cohort** | | | **Validation cohort** | | | |
| --- | --- | --- | --- | --- | --- | --- | --- |
|  | **non-PE**  **(n=24,074)** | **PE**  **(n=1635)** | ***p*-value** | **non-PE**  **(n=1602)** | **PE**  **(n=158)** | ***p*-value** | |
| Age, y^a^ | 31.34 (4.64) | 32.84 (5.15) | *** | 33.21 (4.53) | 34.11 (4.45) | 0.02 | |
| Height, cm^a^ | 158.91 (4.92) | 157.84 (4.9) | *** | 158.04 (5.05) | 158.34 (5.15) | 0.48 | |
| Pre-gestational BMI, kg/m^2a^ | 21.24 (2.93) | 22.86 (3.56) | *** | 22.03 (3.08) | 24.06 (4.05) | *** | |
| Gravidity^a^ | 2.34 (1.33) | 2.44 (1.43) | *** | 2.57 (1.46) | 2.22 (1.22) | *** | |
| NVP |  |  | *** |  |  | 0.0455 | |
| No | 2241 (9.31%) | 230 (14.07%) |  | 242 (15.11%) | 34 (21.52%) |  | |
| Yes | 21833 (90.69%) | 1405 (85.93%) |  | 1360 (84.89%) | 124 (78.48%) |  | |
| Menstrual cycle irregularity |  |  | *** |  |  | 0.0571 | |
| No | 22166 (92.07%) | 1449 (88.62%) |  | 1442 (90.01%) | 134 (84.81%) |  | |
| Yes | 1908 (7.93%) | 186 (11.38%) |  | 160 (9.99%) | 24 (15.19%) |  |  |
| Multifetal pregnancy |  |  | *** |  |  | *** | |
| No | 23277 (96.69%) | 1444 (88.32%) |  | 1509 (94.19%) | 135 (85.44%) |  | |
| Yes | 797 (3.31%) | 191 (11.68%) |  | 93 (5.81%) | 23 (14.56%) |  | |
| Assisted reproductive technology |  |  | *** |  |  | 0.1315 | |
| No | 22688 (94.24%) | 1455 (88.99%) |  | 1454 (90.76%) | 137 (86.71%) |  | |
| Yes | 1386 (5.76%) | 180 (11.01%) |  | 148 (9.24%) | 21 (13.29%) |  | |
| Chronic hypertension |  |  | *** |  |  | *** | |
| No | 23967 (99.56%) | 1412 (86.36%) |  | 1584 (98.88%) | 114 (72.15%) |  | |
| Yes | 107 (0.44%) | 223 (13.64%) |  | 18 (1.12%) | 44 (27.85%) |  | |
| Previous PE |  |  | *** |  |  | *** | |
| No | 24002 (99.7%) | 1594 (97.49%) |  | 1591 (99.31%) | 143 (90.51%) |  | |
| Yes | 72 (0.3%) | 41 (2.51%) |  | 11 (0.69%) | 15 (9.49%) |  | |
| Previous stillbirth |  |  | *** |  |  | 0.283 | |
| No | 23385 (97.14%) | 1563 (95.6%) |  | 1526 (95.26%) | 154 (97.47%) |  | |
| Yes | 689 (2.86%) | 72 (4.4%) |  | 76 (4.74%) | 4 (2.53%) |  | |
| Pre-gestational diabetes |  |  | *** |  |  | *** | |
| No | 23906 (99.3%) | 1604 (98.1%) |  | 1589 (99.19%) | 147 (93.04%) |  | |
| Yes | 168 (0.7%) | 31 (1.9%) |  | 13 (0.81%) | 11 (6.96%) |  | |
| Previous miscarriage |  |  | *** |  |  | 0.718 | |
| No | 22503 (93.47%) | 1491 (91.19%) |  | 1440 (89.89%) | 144 (91.14%) |  | |
| Yes | 1571 (6.53%) | 144 (8.81%) |  | 162 (10.11%) | 14 (8.86%) |  | |
| Scarred uterus |  |  | *** |  |  | *** | |
| No | 18108 (75.22%) | 1344 (82.2%) |  | 1033 (64.48%) | 136 (86.08%) |  | |
| Yes | 5966 (24.78%) | 291 (17.8%) |  | 569 (35.52%) | 22 (13.92%) |  | |
| Chronic renal disease |  |  | *** |  |  | 0.7632 | |
| No | 23268 (96.65%) | 1441 (88.13%) |  | 1432 (89.39%) | 143 (90.51%) |  | |
| Yes | 806 (3.35%) | 194 (11.87%) |  | 170 (10.61%) | 15 (9.49%) |  | |
| Family history of hypertension |  |  | *** |  |  | *** | |
| No | 23078 (95.86%) | 1478 (90.4%) |  | 1550 (96.75%) | 139 (87.97%) |  | |
| Yes | 996 (4.14%) | 157 (9.6%) |  | 52 (3.25%) | 19 (12.03%) |  | |

**^a^** Data are presented as mean (standard deviation); ***: *p*-value < 0.001.

*PE* Preeclampsia, *BMI* Body Mass Index, *NVP* Nausea and Vomiting in Pregnancy.
