# Supplementary material for: An early screening model for preeclampsia: utilizing zero-cost maternal predictors exclusively
Source: Hypertens Res. 2024 Feb 7;47(4):1051–62. doi: 10.1038/s41440-023-01573-8 (PMC10994845; doi:10.1038/s41440-023-01573-8)
Supplement: Supplementary file 3 — Supplementary Table 3 [file 41440_2023_1573_MOESM3_ESM.docx]

### Supplemental Table 3. Model metrics for internal validation.

| **Model** | **AUC** | **Sensitivity** | **Specificity** | **PPV** | **NPV** | **F1** | **Accuracy** | **Brier score** | **Kappa** | **MCC** |
| --- | --- | --- | --- | --- | --- | --- | --- | --- | --- | --- |
| **AdaBoost** | 0.8775 (0.8612, 0.8942) | 0.7271 (0.6924, 0.7619) | 0.9008 (0.9000, 0.9023) | 0.7011 (0.6771, 0.7206) | 0.9127 (0.9016, 0.9233) | 0.7153 (0.6866, 0.7364) | 0.8595 (0.8506, 0.8678) | 0.2218 (0.2207, 0.2232) | 0.6215 (0.5900, 0.6467) | 0.6218 (0.5901, 0.6475) |
| **RF** | 0.8727 (0.8516, 0.8848) | 0.7161 (0.6742, 0.7465) | 0.9008 (0.9000, 0.9023) | 0.695 (0.6718, 0.7175) | 0.9066 (0.8942, 0.9183) | 0.702 (0.6773, 0.7276) | 0.8544 (0.8458, 0.8636) | 0.0904 (0.0836, 0.0985) | 0.6057 (0.5735, 0.6337) | 0.6058 (0.5736, 0.6341) |
| **MLP** | 0.8604 (0.8443, 0.8785) | 0.6640 (0.6311, 0.7061) | 0.9008 (0.9000, 0.9023) | 0.6825 (0.6592, 0.7054) | 0.8951 (0.8810, 0.9070) | 0.6756 (0.6479, 0.7059) | 0.8447 (0.8338, 0.8533) | 0.1061 (0.0991, 0.1154) | 0.5673 (0.5355, 0.5958) | 0.5675 (0.5358, 0.5958) |
| **GBDT** | 0.8565 (0.8367, 0.8796) | 0.6877 (0.6436, 0.7218) | 0.9007 (0.9000, 0.9023) | 0.6866 (0.6625, 0.7122) | 0.8989 (0.8855, 0.9115) | 0.6844 (0.6556, 0.7141) | 0.8479 (0.8383, 0.8579) | 0.0937 (0.0856, 0.1022) | 0.5885 (0.5572, 0.6199) | 0.5886 (0.5573, 0.6201) |
| **GNB** | 0.8535 (0.8330, 0.8747) | 0.6751 (0.6383, 0.7168) | 0.9008 (0.9000, 0.9026) | 0.6861 (0.6636, 0.7069) | 0.8983 (0.8850, 0.9107) | 0.6832 (0.6526, 0.7075) | 0.8475 (0.8368, 0.8564) | 0.1486 (0.1376, 0.1624) | 0.5821 (0.5459, 0.6102) | 0.5822 (0.5461, 0.6103) |
| **XGBoost** | 0.8730 (0.8525, 0.8904) | 0.7177 (0.6802, 0.7546) | 0.9007 (0.9000, 0.9027) | 0.6974 (0.6731, 0.7213) | 0.909 (0.8964, 0.9209) | 0.7072 (0.6807, 0.7331) | 0.8564 (0.8477, 0.8656) | 0.0878 (0.0809, 0.0959) | 0.6071 (0.5749, 0.6349) | 0.6072 (0.5750, 0.6352) |
| **LR** | 0.8528 (0.8240, 0.8714) | 0.6530 (0.6159, 0.6950) | 0.9006 (0.9000, 0.9020) | 0.6786 (0.6550, 0.7007) | 0.892 (0.8781, 0.9037) | 0.6683 (0.6383, 0.6956) | 0.842 (0.8317, 0.8512) | 0.1097 (0.1025, 0.1197) | 0.558 (0.5239, 0.5899) | 0.5583 (0.5248, 0.5899) |
| **SVM** | 0.8348 (0.8193, 0.8636) | 0.6672 (0.6249, 0.7118) | 0.9007 (0.9001, 0.9017) | 0.6817 (0.6572, 0.7052) | 0.8947 (0.8795, 0.9068) | 0.6745 (0.6438, 0.7069) | 0.8443 (0.8338, 0.8543) | 0.1096 (0.1023, 0.1184) | 0.5698 (0.5320, 0.6090) | 0.57 (0.5322, 0.6091) |
| **CatBoost** | 0.8724 (0.8511, 0.8921) | 0.7129 (0.6695, 0.7461) | 0.9007 (0.9000, 0.9026) | 0.6951 (0.6700, 0.7192) | 0.9069 (0.8939, 0.9185) | 0.7026 (0.6734, 0.7288) | 0.8547 (0.8456, 0.8642) | 0.0893 (0.0816, 0.0973) | 0.6064 (0.5759, 0.6341) | 0.6065 (0.5759, 0.6345) |
| **LightGBM** | 0.8565 (0.8421, 0.8758) | 0.6767 (0.6388, 0.7155) | 0.9008 (0.9000, 0.9025) | 0.6851 (0.6601, 0.7110) | 0.8974 (0.8838, 0.9100) | 0.6811 (0.6544, 0.7091) | 0.8467 (0.8370, 0.8562) | 0.0962 (0.0886, 0.1050) | 0.5895 (0.5605, 0.6177) | 0.5896 (0.5606, 0.6178) |

*AUC* Area Under the Receiver Operating Characteristic Curve, *PPV* Positive Predictive Value; *NPV*, Negative Predictive Value, *AdaBoost* Adaptive Boosting, *RF* Random Forest, *MLP* Multi-Layer Perceptron, *GBDT* Gradient Boosting Decision Tree, *GBN* Gaussian Naive Bayes, *XGBoost* Extreme Gradient Boosting, *LR* Logistic Regression, *SVM* Support Vector Machines, *CatBoost* Category Boosting, *LightGBM* Light Gradient Boosted Machine, *MCC* Matthew's Correlation Coefficient.
