# Supplementary material for: An early screening model for preeclampsia: utilizing zero-cost maternal predictors exclusively
Source: Hypertens Res. 2024 Feb 7;47(4):1051–62. doi: 10.1038/s41440-023-01573-8 (PMC10994845; doi:10.1038/s41440-023-01573-8)
Supplement: Supplementary file 4 — Supplementary Table 4 [file 41440_2023_1573_MOESM4_ESM.docx]

### Supplemental Table 4. Model metrics for external validation.

| **Model** | **AUC** | **Sensitivity** | **Specificity** | **PPV** | **NPV** | **F1** | **Accuracy** | **Brier score** | **Kappa** | **MCC** |
| --- | --- | --- | --- | --- | --- | --- | --- | --- | --- | --- |
| **AdaBoost** | 0.8008 | 0.5190 | 0.9014 | 0.3389 | 0.9494 | 0.4081 | 0.8665 | 0.2323 | 0.3402 | 0.3489 |
| **RF** | 0.7850 | 0.5027 | 0.9032 | 0.3352 | 0.9485 | 0.400 | 0.8642 | 0.0807 | 0.335 | 0.3444 |
| **MLP** | 0.7719 | 0.481 | 0.9101 | 0.3455 | 0.9468 | 0.4021 | 0.8716 | 0.1131 | 0.3323 | 0.3381 |
| **GBDT** | 0.781 | 0.462 | 0.9001 | 0.3133 | 0.9443 | 0.3734 | 0.8608 | 0.0873 | 0.2983 | 0.3055 |
| **GNB** | 0.725 | 0.4747 | 0.9026 | 0.3247 | 0.9457 | 0.3856 | 0.8642 | 0.2266 | 0.3123 | 0.3194 |
| **XGBoost** | 0.7874 | 0.5063 | 0.9001 | 0.3333 | 0.9487 | 0.402 | 0.8648 | 0.0795 | 0.3294 | 0.3386 |
| **LR** | 0.7506 | 0.4873 | 0.9107 | 0.35 | 0.9474 | 0.4074 | 0.8727 | 0.1087 | 0.3383 | 0.3441 |
| **SVM** | 0.7267 | 0.443 | 0.9032 | 0.3111 | 0.9427 | 0.3655 | 0.8619 | 0.1027 | 0.2907 | 0.2964 |
| **CatBoost** | 0.7819 | 0.5 | 0.902 | 0.3347 | 0.9482 | 0.401 | 0.8659 | 0.0824 | 0.3288 | 0.3372 |
| **LightGBM** | 0.7624 | 0.5 | 0.9039 | 0.3391 | 0.9483 | 0.4041 | 0.8676 | 0.0851 | 0.3327 | 0.3406 |

*AUC* Area Under the Receiver Operating Characteristic Curve, *PPV* Positive Predictive Value; *NPV*, Negative Predictive Value, *AdaBoost* Adaptive Boosting, *RF* Random Forest, *MLP* Multi-Layer Perceptron, *GBDT* Gradient Boosting Decision Tree, *GBN* Gaussian Naive Bayes, *XGBoost* Extreme Gradient Boosting, *LR* Logistic Regression, *SVM* Support Vector Machines, *CatBoost* Category Boosting, *LightGBM* Light Gradient Boosted Machine, *MCC* Matthew's Correlation Coefficient.
