# Supplementary material for: An early screening model for preeclampsia: utilizing zero-cost maternal predictors exclusively
Source: Hypertens Res. 2024 Feb 7;47(4):1051–62. doi: 10.1038/s41440-023-01573-8 (PMC10994845; doi:10.1038/s41440-023-01573-8)
Supplement: Supplementary file 5 — Supplementary Table 5 [file 41440_2023_1573_MOESM5_ESM.docx]

### Supplemental Table 5. Performance Metrics of the AdaBoost Model for All-PE, Preterm-PE, and Early Onset-PE Prediction in External Validation.

|  | **AUC** | **Sensitivity** | **Specificity** | **PPV** | **NPV** | **F1** | **Accuracy** | **Brier score** | **Kappa** | **MCC** |
| --- | --- | --- | --- | --- | --- | --- | --- | --- | --- | --- |
| **all-PE** | 0.8008 | 0.5190 | 0.9014 | 0.3389 | 0.9494 | 0.4081 | 0.8665 | 0.2323 | 0.3402 | 0.3489 |
| **preterm-PE** | 0.8164 | 0.5323 | 0.9058 | 0.1710 | 0.9815 | 0.2588 | 0.8926 | 0.2317 | 0.2148 | 0.2566 |
| **early onset-PE** | 0.8215 | 0.5815 | 0.9302 | 0.097 | 0.9914 | 0.1615 | 0.9233 | 0.2317 | 0.1395 | 0.1908 |

*AUC* Area Under the Receiver Operating Characteristic Curve, *PPV* Positive Predictive Value; *NPV*, Negative Predictive Value, *AdaBoost* Adaptive Boosting, *RF* Random Forest, *MLP* Multi-Layer Perceptron, *GBDT* Gradient Boosting Decision Tree, *GBN* Gaussian Naive Bayes, *XGBoost* Extreme Gradient Boosting, *LR* Logistic Regression, *SVM* Support Vector Machines, *CatBoost* Category Boosting, *LightGBM* Light Gradient Boosted Machine, *MCC* Matthew's Correlation Coefficient.
