# Supplementary material for: An early screening model for preeclampsia: utilizing zero-cost maternal predictors exclusively
Source: Hypertens Res. 2024 Feb 7;47(4):1051–62. doi: 10.1038/s41440-023-01573-8 (PMC10994845; doi:10.1038/s41440-023-01573-8)
Supplement: Supplementary file 6 — Supplementary Table 6 [file 41440_2023_1573_MOESM6_ESM.docx]

### Supplemental Table 6. Comparative Performance Metrics of the AdaBoost Model in External Validation Across Full Cohort, Chronic Hypertension Subgroup, and Non-Chronic Hypertension Subgroup.

|  | **AUC** | **Sensitivity** | **Specificity** | **PPV** | **NPV** | **F1** | **Accuracy** | **Brier score** | **Kappa** | **MCC** |
| --- | --- | --- | --- | --- | --- | --- | --- | --- | --- | --- |
| **all-PE** | 0.8008 | 0.5190 | 0.9014 | 0.3389 | 0.9494 | 0.4081 | 0.8665 | 0.2323 | 0.3402 | 0.3489 |
| **Chronic Hypertension** | 0.6564 | 0.3414 | 0.7224 | 0.1710 | 0.7502 | 0.4690 | 0.4521 | 0.2842 | 0.1942 | 0.2230 |
| **Non-Chronic Hypertension** | 0.7571 | 0.4392 | 0.8952 | 0.2312 | 0.9572 | 0.3032 | 0.8652 | 0.2517 | 0.1395 | 0.1062 |

*AUC* Area Under the Receiver Operating Characteristic Curve, *PPV* Positive Predictive Value; *NPV*, Negative Predictive Value, *AdaBoost* Adaptive Boosting, *RF* Random Forest, *MLP* Multi-Layer Perceptron, *GBDT* Gradient Boosting Decision Tree, *GBN* Gaussian Naive Bayes, *XGBoost* Extreme Gradient Boosting, *LR* Logistic Regression, *SVM* Support Vector Machines, *CatBoost* Category Boosting, *LightGBM* Light Gradient Boosted Machine, *MCC* Matthew's Correlation Coefficient.
