# Supplementary material for: An early screening model for preeclampsia: utilizing zero-cost maternal predictors exclusively
Source: Hypertens Res. 2024 Feb 7;47(4):1051–62. doi: 10.1038/s41440-023-01573-8 (PMC10994845; doi:10.1038/s41440-023-01573-8)
Supplement: Supplementary file 7 — Supplementary Table 7 [file 41440_2023_1573_MOESM7_ESM.docx]

### Supplemental Table 7. Lab test results of the training cohort.

| **Feature** | **non-PE (n=24,074)** | | **PE (n=1635)** | | ***p*-value** |
| --- | --- | --- | --- | --- | --- |
|  | **Normal** | **Abnormal** | **Normal** | **Abnormal** |  |
| **Urinary Vitamin C** | 10508 | 0 | 669 | 0 | - |
| **International Normalized Ratio (INR)** | 14903 | 11 | 840 | 0 | 0.4311 |
| **Red Blood Cell Distribution Width CV (RDW-CV)** | 13000 | 1682 | 717 | 117 | 0.024 |
| **Red Blood Cell Distribution Width SD (RDW-SD)** | 14258 | 415 | 798 | 36 | 0.0129 |
| **Rh Blood Type** | 589 | 8906 | 13 | 412 | 0.0079 |
| **Gamma-Glutamyl Transferase (GGT)** | 14399 | 122 | 787 | 16 | *** |
| **Albumin** | 12286 | 2231 | 667 | 136 | 0.2313 |
| **Albumin/Globulin Ratio (A/G ratio)** | 13646 | 871 | 740 | 63 | 0.0333 |
| **Vaginal Fluid Leukocytes** | 10123 | 3711 | 581 | 205 | 0.6469 |
| **Vaginal Fluid Sialidase** | 4519 | 9407 | 296 | 525 | 0.0324 |
| **White Blood Cell Count (WBC)** | 10875 | 3408 | 499 | 289 | *** |
| **Urinary Leukocyte Microscopy** | 12963 | 871 | 740 | 46 | 0.6178 |
| **Alanine Aminotransferase (ALT)** | 14127 | 394 | 775 | 28 | 0.1922 |
| **Hepatitis C Antibody** | 14173 | 15 | 781 | 1 | 0.8536 |
| **Urinary Calcium Oxalate Crystals** | 14188 | 61 | 828 | 5 | 0.4644 |
| **Thyroid Stimulating Hormone (TSH)** | 11726 | 3150 | 591 | 241 | *** |
| **Large Platelet Ratio** | 14170 | 55 | 815 | 1 | 0.2284 |
| **Monoamine Oxidase** | 14913 | 9 | 850 | 1 | 0.5191 |
| **Herpes Simplex Virus Type II IgG** | 8493 | 1343 | 538 | 108 | 0.0289 |
| **Herpes Simplex Virus Type II IgM** | 13651 | 39 | 738 | 1 | 0.4513 |
| **Monocyte Ratio** | 12065 | 2618 | 667 | 167 | 0.1082 |
| **Monocyte Count** | 13185 | 1498 | 690 | 144 | *** |
| **Cholinesterase** | 14786 | 136 | 844 | 7 | 0.7903 |
| **High Immature Reticulocyte Fraction** | 3784 | 9801 | 121 | 630 | *** |
| **Low Nucleic Acid Reticulocyte Percentage** | 3687 | 9929 | 119 | 633 | *** |
| **Low-Density Lipoprotein Cholesterol (LDL-C)** | 11892 | 1215 | 700 | 93 | 0.0213 |
| **Rubella Virus IgG Antibody** | 1172 | 8928 | 71 | 599 | 0.4296 |
| **Rubella Virus IgM Antibody** | 13960 | 18 | 774 | 2 | 0.3419 |
| **Triglycerides** | 10370 | 2328 | 515 | 230 | *** |
| **High Nucleic Acid Reticulocyte Percentage** | 12269 | 1347 | 640 | 112 | *** |
| **High-Density Lipoprotein Cholesterol (HDL-C)** | 2628 | 10479 | 247 | 546 | *** |
| **Toxoplasma gondii IgG Antibody** | 9348 | 752 | 609 | 61 | 0.1155 |
| **Toxoplasma gondii IgM Antibody** | 13948 | 8 | 763 | 0 | 0.5083 |
| **Urinary Leukocyte Esterase** | 13812 | 70 | 809 | 5 | 0.6686 |
| **Red Blood Cell Count (RBC)** | 13899 | 384 | 764 | 24 | 0.5476 |
| **Urinary Erythrocyte Microscopy** | 13574 | 261 | 760 | 26 | 0.0052 |
| **Hematocrit** | 12556 | 2129 | 747 | 87 | 0.0011 |
| **Jaundice Index** | 4630 | 5 | 447 | 0 | 0.4872 |
| **Activated Partial Thromboplastin Time (APTT)** | 14735 | 186 | 830 | 10 | 0.8865 |
| **Creatinine** | 10762 | 3707 | 621 | 172 | 0.0133 |
| **Thyroid Peroxidase Antibodies (TPOAb)** | 8979 | 1167 | 393 | 73 | 0.0062 |
| **Indirect Bilirubin** | 4915 | 10005 | 244 | 607 | 0.0098 |
| **Alkaline Phosphatase (ALP)** | 12312 | 2208 | 735 | 68 | *** |
| **Cytomegalovirus IgG Antibody** | 201 | 9899 | 15 | 655 | 0.6565 |
| **Cytomegalovirus IgM Antibody** | 13929 | 27 | 760 | 3 | 0.2336 |
| **Anti-Alkaline Hemoglobin** | 10017 | 185 | 586 | 6 | 0.1513 |
| **Urinary Granular Casts** | 14251 | 1 | 833 | 0 | 0.8090 |
| **Urinary Waxy Casts** | 14138 | 0 | 824 | 0 | - |
| **Granulocyte Ratio** | 8246 | 6437 | 458 | 376 | 0.4813 |
| **Granulocyte Count** | 10905 | 3778 | 533 | 301 | *** |
| **Lymphocyte Ratio** | 10159 | 4524 | 543 | 291 | 0.0132 |
| **Lymphocyte Count** | 14649 | 34 | 832 | 2 | 0.9616 |
| **Urinary Phosphate Crystals** | 14175 | 77 | 830 | 3 | 0.4866 |
| **Urinary Squamous Epithelial Cells** | 14060 | 192 | 817 | 16 | 0.1676 |
| **Treponema Pallidum Particle Agglutination (TPPA)** | 11741 | 0 | 531 | 0 | - |
| **Aspartate Aminotransferase (AST)** | 14276 | 245 | 784 | 19 | 0.1501 |
| **Urine Specific Gravity** | 12462 | 1371 | 706 | 80 | 0.8076 |
| **Urobilinogen** | 13615 | 219 | 777 | 9 | 0.3350 |
| **Urinary Bilirubin** | 12926 | 908 | 747 | 39 | 0.0759 |
| **Proteinuria** | 11692 | 2142 | 641 | 145 | 0.0261 |
| **Urinary Occult Blood** | 12094 | 1740 | 673 | 113 | 0.1403 |
| **Urea** | 5985 | 8484 | 342 | 451 | 0.3265 |
| **Uric Acid** | 14437 | 32 | 790 | 3 | 0.3677 |
| **Urinary Uric Acid Crystals** | 14244 | 8 | 832 | 1 | 0.4628 |
| **Thrombin Time** | 14766 | 150 | 834 | 6 | 0.4067 |
| **Vaginal Discharge Pus Cells** | 13766 | 116 | 814 | 0 | 0.0088 |
| **Urinary Pus Cells Microscopy** | 13793 | 42 | 783 | 3 | 0.7006 |
| **Mean Corpuscular Hemoglobin (MCH)** | 11486 | 2797 | 604 | 184 | 0.0097 |
| **Mean Corpuscular Hemoglobin Concentration (MCHC)** | 12683 | 1600 | 678 | 110 | 0.0175 |
| **Mean Corpuscular Volume (MCV)** | 12509 | 2174 | 667 | 167 | *** |
| **Mean Platelet Volume (MPV)** | 14199 | 28 | 814 | 2 | 0.7636 |
| **Glucose** | 14130 | 691 | 769 | 54 | 0.0128 |
| **Urinary Yeast-like Fungus** | 9783 | 0 | 461 | 0 | - |
| **Prealbumin** | 13861 | 654 | 786 | 17 | 0.0013 |
| **Urine Clarity** | 13869 | 11 | 814 | 0 | 0.4217 |
| **Globulin** | 14614 | 312 | 837 | 14 | 0.3745 |
| **Free Triiodothyronine (FT3)** | 14268 | 40 | 817 | 3 | 0.6517 |
| **Human Immunodeficiency Virus Antibody (HIV Antibody)** | 10161 | 0 | 407 | 0 | - |
| **Hemolysis Index** | 4620 | 15 | 447 | 0 | 0.2284 |
| **Basophil Ratio** | 14683 | 0 | 834 | 0 | - |
| **Basophil Count** | 14683 | 0 | 834 | 0 | - |
| **Eosinophil Ratio** | 12757 | 1926 | 719 | 115 | 0.5766 |
| **Eosinophil Count** | 14651 | 32 | 833 | 1 | 0.55 |
| **Urinary pH** | 13218 | 615 | 761 | 25 | 0.0917 |
| **Ferritin** | 13382 | 635 | 802 | 45 | 0.2898 |
| **Ketone Bodies** | 11983 | 1851 | 674 | 112 | 0.4868 |
| **Urine Transparency** | 13835 | 0 | 786 | 0 | - |
| **Hyaline Casts** | 14251 | 1 | 832 | 1 | 0.0059 |
| **Reticulocyte Percentage** | 4755 | 9201 | 172 | 622 | *** |
| **Reticulocyte Absolute Count** | 8853 | 5103 | 361 | 433 | *** |
| **Trace Elements Calcium** | 11422 | 1937 | 562 | 107 | 0.2850 |
| **Trace Elements Magnesium** | 13277 | 82 | 665 | 4 | 0.9590 |
| **Trace Elements Iron** | 11067 | 2292 | 554 | 115 | 0.9824 |
| **Trace Elements Copper** | 12123 | 1236 | 615 | 54 | 0.3025 |
| **Trace Elements Zinc** | 12205 | 1154 | 622 | 47 | 0.1456 |
| **Vitamin A** | 4384 | 0 | 377 | 0 | - |
| **Vitamin C** | 8103 | 1481 | 550 | 104 | 0.7586 |
| **Vitamin E** | 4384 | 0 | 377 | 0 | - |
| **Bacterial Vaginosis** | 11478 | 2006 | 700 | 68 | *** |
| **Adenosine Deaminase** | 14874 | 47 | 847 | 4 | 0.4384 |
| **Urinary Small Round Epithelial Cells** | 14247 | 5 | 833 | 0 | 0.5887 |
| **Hemoglobin** | 13543 | 740 | 755 | 33 | 0.2186 |
| **Hemoglobin A** | 8097 | 2105 | 449 | 143 | 0.0402 |
| **Hemoglobin A2** | 8977 | 1225 | 506 | 86 | 0.0681 |
| **Hemoglobin Bart's** | 10202 | 0 | 592 | 0 | - |
| **Hemoglobin C** | 10202 | 0 | 592 | 0 | - |
| **Hemoglobin CS** | 10202 | 0 | 592 | 0 | - |
| **Hemoglobin D** | 9777 | 0 | 559 | 0 | - |
| **Hemoglobin E** | 10202 | 0 | 592 | 0 | - |
| **Hemoglobin F** | 9598 | 180 | 553 | 6 | 0.1843 |
| **Hemoglobin H** | 10202 | 0 | 592 | 0 | - |
| **Hemoglobin J** | 10202 | 0 | 592 | 0 | - |
| **Hemoglobin S** | 10202 | 0 | 592 | 0 | - |
| **Hemoglobin Fast Fraction** | 10202 | 0 | 592 | 0 | - |
| **Hemoglobin Slow Fraction** | 10202 | 0 | 592 | 0 | - |
| **Plasma D-Dimer** | 8286 | 5621 | 526 | 243 | *** |
| **Plasma Prothrombin Time (PT)** | 14886 | 31 | 839 | 1 | 0.5782 |
| **Plasma Fibrinogen** | 6034 | 8896 | 271 | 569 | *** |
| **Serum Alpha-L-Fucosidase** | 14388 | 125 | 788 | 15 | 0.0035 |
| **Plateletcrit** | 11389 | 2838 | 537 | 279 | *** |
| **Platelet Count** | 14112 | 171 | 764 | 24 | *** |
| **Platelet Distribution Width (PDW)** | 3608 | 10619 | 166 | 650 | 0.0013 |
| **Urinary Nitrites** | 13718 | 116 | 778 | 8 | 0.5939 |
| **Urine Color** | 13835 | 0 | 786 | 0 | - |
| **Hepatitis B e Antibody (quantitative)** | 7475 | 3475 | 351 | 161 | 0.8904 |
| **Hepatitis B e Antigen (quantitative)** | 10729 | 221 | 499 | 13 | 0.4154 |
| **Hepatitis B Surface Antibody (quantitative)** | 2741 | 8209 | 131 | 381 | 0.7774 |
| **Hepatitis B Surface Antigen (quantitative)** | 13250 | 1253 | 755 | 65 | 0.4788 |
| **Hepatitis B Core Antibody (quantitative)** | 5982 | 4968 | 263 | 249 | 0.1473 |
| **Trichomonas vaginalis** | 13470 | 18 | 768 | 2 | 0.3625 |
| **Free Thyroxine (FT4)** | 14439 | 428 | 787 | 45 | *** |
| **Free Triiodothyronine (FT3)** | 14091 | 391 | 747 | 39 | *** |
| **Nucleated Red Blood Cell Percentage** | 4161 | 297 | 398 | 28 | 0.9436 |
| **Nucleated Red Blood Cell Count** | 4161 | 297 | 398 | 28 | 0.9436 |
| **Apolipoprotein A-I (ApoA-I)** | 7640 | 5058 | 495 | 250 | *** |
| **Apolipoprotein A-I/B** | 12681 | 17 | 742 | 3 | 0.0643 |
| **Apolipoprotein B (ApoB)** | 11587 | 1517 | 705 | 88 | 0.6816 |
| **Fungal Spores** | 12552 | 1330 | 743 | 71 | 0.4176 |
| **Fungal Hyphae** | 12779 | 709 | 734 | 36 | 0.4809 |
| **Lipid Profile** | 4628 | 5 | 447 | 0 | 0.4871 |
| **Direct Bilirubin** | 14891 | 33 | 850 | 1 | 0.5261 |
| **Intermediate Nucleic Acid Reticulocyte Percentage** | 4100 | 9516 | 142 | 610 | *** |
| **Total Cholesterol** | 9886 | 2813 | 561 | 184 | 0.1046 |
| **Total Bilirubin** | 10750 | 3766 | 558 | 245 | 0.0041 |
| **Total Bile Acids** | 14672 | 250 | 842 | 9 | 0.1678 |
| **Total Protein** | 13829 | 688 | 770 | 33 | 0.4121 |
| **Total Carbon Dioxide** | 12147 | 181 | 556 | 8 | 0.9234 |
| **Other Findings in Urine** | 13835 | 0 | 786 | 0 | - |

The count for each category is provided in the table. Data that are missing are not listed in the table; ***: *p*-value < 0.001.

*PE* Preeclampsia.
