# Supplementary figures and images for: An early screening model for preeclampsia: utilizing zero-cost maternal predictors exclusively
Source: Hypertens Res. 2024 Feb 7;47(4):1051–62. doi: 10.1038/s41440-023-01573-8 (PMC10994845; doi:10.1038/s41440-023-01573-8)

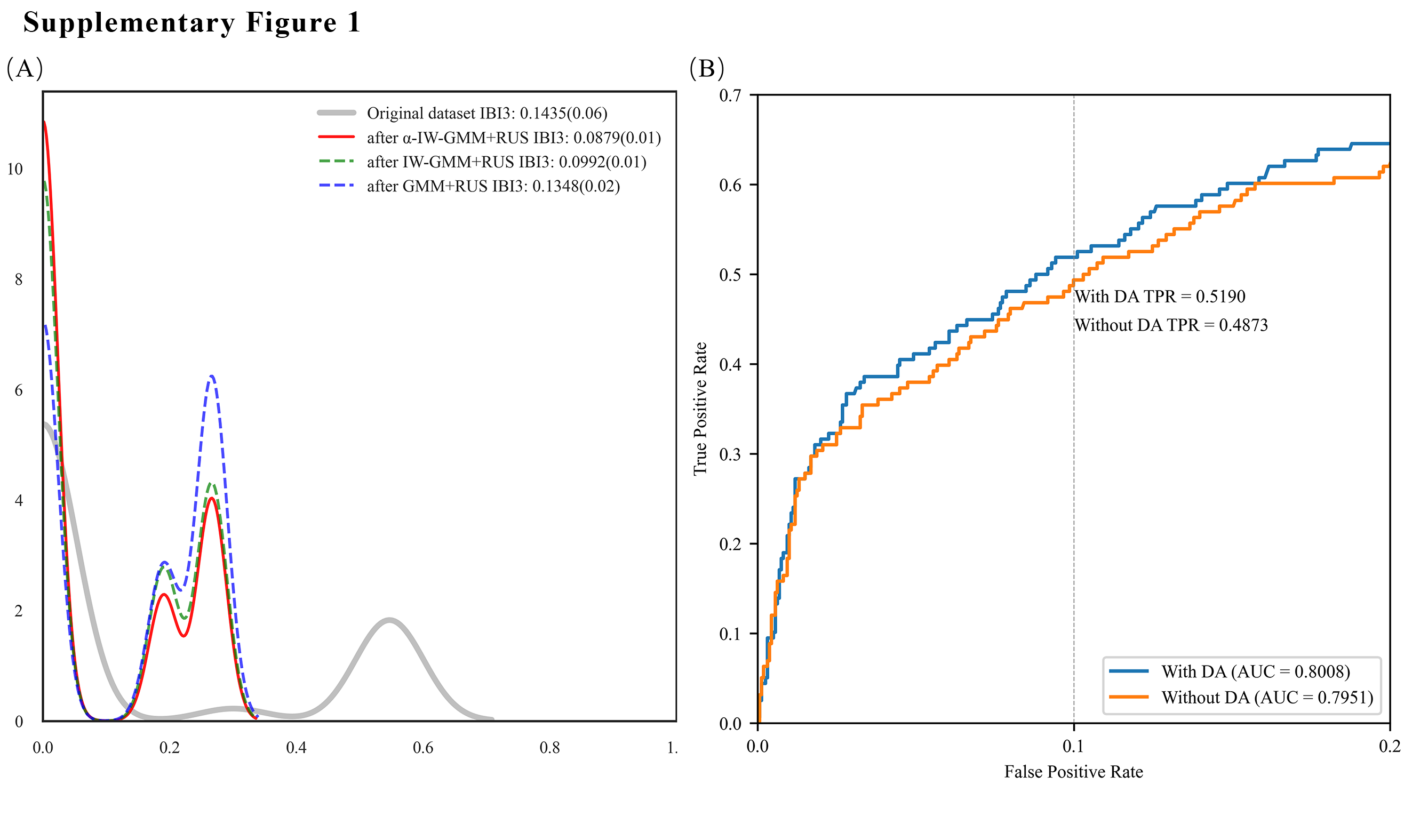

Supplement: Supplementary file 8 — Supplementary Figure 1 [file 41440_2023_1573_MOESM8_ESM.tif]

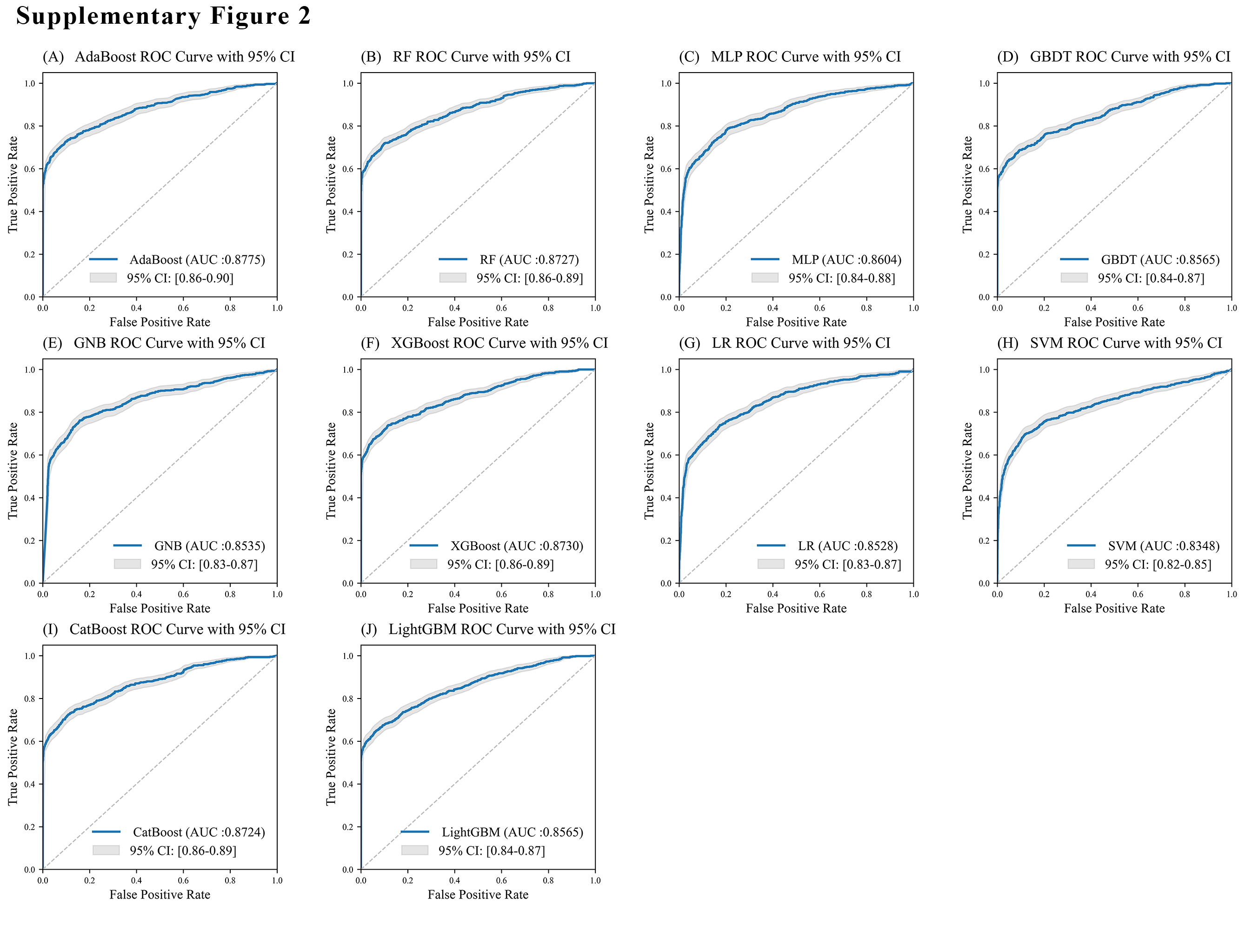

Supplement: Supplementary file 9 — Supplementary Figure 2 [file 41440_2023_1573_MOESM9_ESM.tif]
