# Supplementary material for: An early screening model for preeclampsia: utilizing zero-cost maternal predictors exclusively
Source: Hypertens Res. 2024 Feb 7;47(4):1051–62. doi: 10.1038/s41440-023-01573-8 (PMC10994845; doi:10.1038/s41440-023-01573-8)
Supplement: Supplementary file 10 — Supplementary Figure legend [file 41440_2023_1573_MOESM10_ESM.docx]

### Supplementary Figure 1: Evaluation of different DA methods and their impact on model performance.

(**A**). A density distribution of IBI^3^ values for all positive samples before and after the implementation of three different DA methods: α-inverse weighted GMM+RUS, inverse weighted GMM+RUS, and GMM+RUS. Prior to DA, the IBI^3^ values for positive samples in the original dataset concentrate around two peaks, IBI^3^=0 and IBI^3^=0.55, with an average BI^3^ value of 0.1435. Following DA, all three methods bring the IBI^3^ values closer to 0, enhancing the distribution. Among these methods, α-inverse weighted GMM+RUS is the most effective, achieving the lowest BI^3^ value of 0.0879; **(B)**. The AUC curve for the AdaBoost model on the external validation set, before (AUC = 0.7951, Sensitivity at 10% FPR = 0.4873) and after (AUC = 0.8008, Sensitivity at 10% FPR = 0.5190) DA.

### Supplementary Figure 2: Evaluation of different DA methods and their impact on model performance.

Figures **(A)-(J)** display the individual ROC curves of each model, including their corresponding 95% CIs, and it can be clearly observed that, while all models perform well, the ensemble models (such as AdaBoost, XGBoost and CatBoost) are particularly outstanding.
